# Ultrasonic Nonlinearity Experiment due to Plastic Deformation of Aluminum Plate Due to Bending Damage

**DOI:** 10.3390/ma16124241

**Published:** 2023-06-08

**Authors:** Junpil Park, Mohammed Aslam, Jaesun Lee

**Affiliations:** 1 Extreme Environment Design and Manufacturing Engineering, Changwon National University, Changwon 51140, Republic of Korea; junpil@changwon.ac.kr (J.P.); aslam@changwon.ac.kr (M.A.); 2 School of Mechanical Engineering, Changwon National University, Changwon 51140, Republic of Korea

**Keywords:** non-destructive testing, ultrasonic, nonlinearity, lamb wave, bending damage

## Abstract

The nonlinear ultrasonic evaluation technique is useful for assessing micro-defects and microstructure changes caused by fatigue or bending damage. In particular, the guided wave is advantageous for long-distance testing such as piping and plate. Despite these advantages, the study of nonlinear guided wave propagation has received relatively less attention compared to bulk wave techniques. Furthermore, there is a lack of research on the correlation between nonlinear parameters and material properties. In this study, the relationship between nonlinear parameters and plastic deformation resulting from bending damage was experimentally investigated using Lamb waves. The findings indicated an increase in the nonlinear parameter for the specimen, which was loaded within the elastic limit. Inversely, regions of maximum deflection in specimens with plastic deformation exhibited a decrease in the nonlinear parameter. This research is expected to be helpful for maintenance technology in the nuclear power plant and aerospace fields that require high reliability and accuracy.

## 1. Introduction

In technology-intensive industries such as aerospace, nuclear power, and petrochemical plants, the precision and reliability of equipment and mechanical systems are of utmost importance. Micro-defects in these structures grow rapidly, often surpassing the stage where they can be easily visualized. Current ultrasonic non-destructive testing methods are unable to diagnose defects prior to the formation of macro-cracks [1]. Recent research shows nonlinear ultrasonic techniques have higher sensitivity in detecting corrosion, microdefects, and changes in microstructure [2,3]. Conventional local inspection methods using bulk waves face limitations when it comes to inspecting large structures such as ships, aircraft, containment liner plates, and bridges. Recognizing this challenge, extensive research is being conducted to develop wide-range testing techniques utilizing induced ultrasonic waves [4,5,6]. In particular, nonlinear Lamb waves are advantageous for long-distance testing due to their unique characteristics and the ability to achieve higher sensitivity through various mode selections.

High-tech industries require materials with high reliability and stability. Nonlinear ultrasonic inspection techniques offer the potential to detect microstructural defects resulting from various factors such as micro-defects, corrosion, and plastic deformation due to fatigue or external loads. However, research on the impact of nonlinearity caused by changes in material properties remains insufficient. To bridge this gap, an experimental investigation utilizing Lamb wave-based inspection methods was conducted. The focus of the study was to explore the relationship between bending damage and the manifestation of nonlinearity in aluminum alloy materials. This research contributes to the understanding of material behavior and holds implications for improving the reliability and performance of materials in high-tech industries.

There has been continuous research and development in the field of nonlinear ultrasonic testing techniques, with a particular focus on studying and comparing the fatigue behavior and nonlinearity trends in various materials [7,8]. In particular, Jacobs L.J. and Qu J. studied the nonlinearity of nickel alloy steels using Rayleigh surface waves [9], and the nonlinearity of aluminum alloys was also investigated using a Lamb wave [10]. Deng et al. employed the second method of perturbation theory to investigate the second harmonics of the shear-horizontal (SH) mode in an isotropic plate featuring two free boundaries [11,12]. In addition, they studied the effects of nonlinearity on the cumulative effect, mode analysis, and phase matching, which are important experimental bases for nonlinear ultrasonic techniques. [13,14]. Li Weibin conducted experiments to characterize the thermal fatigue damage of composite laminates using second harmonic Lamb waves [15] and investigated the effect of material microstructure evolution on the acoustic nonlinear response of ultrasound in rolled copper and brass [16]. In addition, Li W. and Park J. conducted a study to detect micro-cracks in materials using non-contact electromagnetic ultrasonic transducers [17,18]. Lissenden presented a method for detecting local fatigue damage on an aluminum plate by utilizing a PVDF (polyvinylidene difluorine) sensor to simultaneously receive shear-horizontal waves and the secondary Lamb waves they generate. The experimental findings were further validated through finite element simulation [19,20]. Furthermore, there is an active research effort among many scholars to diagnose micro-damage through the utilization of second and third harmonics [21,22,23]. Additionally, researchers are exploring methods to enhance sensitivity by mitigating system nonlinearity [24], as well as investigating the effects of thermal aging on sensitivity [24]. However, it is important to note that these studies primarily focus on micro-damage assessment during the material’s initial manufacturing process and intrinsic properties [25,26,27]. Currently, there is a lack of experimental verification regarding material deformation under conditions such as tension or compression due to bending.

Guided ultrasound has traditionally been employed for non-destructive testing of large areas, primarily focusing on diagnosing defects larger than the macro stage. However, limited research has been conducted on the application of nonlinear ultrasound beyond micro-scale defect detection, fatigue analysis, and nonlinearity studies under uniaxial tensile and compressive loads [28,29]. Plates are extensively utilized in various structures, and while some curved plates are manufactured using molds, many products are formed through bending to achieve the desired curvature. Unfortunately, there is a dearth of research exploring material damage resulting from such bending processes. To address this gap, this study experimentally investigates the relationship between bending damage and nonlinearity in aluminum alloy materials using Lamb waves. The study also employs simulations and theoretical analyses to establish a correlation between yield strength and bending, providing comprehensive verification of these findings. To enhance the reliability of the experiment, various experimental conditions were employed, and the key findings can be summarized as follows:

1. It was experimentally verified that the superimposition effect of nonlinear Lamb waves applied to an aluminum plate;
2. A comparative assessment of induced nonlinearities was conducted when subjecting the aluminum plate to bending loads within both the elastic and plastic regions;
3. The experimental investigation further examined nonlinearity tendencies specific to each mode of the Lamb wave on the aluminum plate subjected to bending loads in the plastic region;
4. A comparative analysis was performed to assess and analyze the induced nonlinearity due to compression plasticity and tensile plasticity in the aluminum plate subjected to bending.

## 2. Nonlinear Ultrasonic Theory

Nonlinear ultrasonic waves have the characteristics of generating high harmonics due to the deterioration of material and the change in plasticity due to fatigue and external forces when an acoustic wave passes through the material. The reason for this harmonic generation is that as the high-order elastic constants increase due to material defects, the stress–strain relationship changes into a nonlinear rather than a linear relationship. In other words, it is difficult to explain the plasticity of the stress–strain curve or materials that do not follow the elastic behavior with the linear ultrasonic theory, and a more accurate behavior can be obtained by applying the nonlinear ultrasonic theory.

Nonlinearity can be categorized into two main types: material nonlinearity and geometric nonlinearity. Material nonlinearity arises from the variations in the states of the constituent crystals within the material, deviating from ideal uniformity. This introduces nonlinearity into the material’s behavior. On the other hand, geometric nonlinearity refers to the nonlinearity that arises due to significant deformations or displacements of the material, where the strain-displacement relationship becomes nonlinear.

The nonlinear ultrasonic equation can be expressed as Equation (1) [30].

(1) $$\begin{array}{l} u_{tt}-c_{l}^{2}u_{aa}=\left(3c_{l}^{2}+C_{111}/\rho \right)u_{a}u_{aa}+\left(c_{l}^{2}+C_{166}/\rho \right)\left(v_{a}v_{aa}+w_{a}w_{aa}\right)\\ v_{tt}-c_{s}^{2}v_{aa}=\left(c_{l}^{2}+C_{166}/\rho \right)\left(u_{a}v_{aa}+v_{a}u_{aa}\right)\\ w_{tt}-c_{s}^{2}w_{aa}=\left(c_{l}^{2}+C_{166}/\rho \right)\left(u_{a}w_{aa}+w_{a}u_{aa}\right) \end{array}$$

In contrast to the linear wave equation, the cubic elastic modulus is introduced as $C_{111,\;}C_{166}$ and the solution of the nonlinear ultrasonic equation can be derived by the perturbation method. If this solution is expanded by the sum of the solution of the first fundamental frequency and the solution of the second harmonic frequency, it can be expressed as Equation (2).

(2) $$\begin{array}{l} u_{tt}^{\left(1\right)}-c_{l}^{2}u_{aa}^{\left(1\right)}=0\\ v_{tt}^{\left(1\right)}-c_{s}^{2}v_{aa}^{\left(1\right)}=0\\ w_{tt}^{\left(1\right)}-c_{s}^{2}w_{aa}^{\left(1\right)}=0 \end{array}$$

Considering only the longitudinal component,

(3) $$u_{tt}^{\left(1\right)}-c_{l}^{2}u_{aa}^{\left(1\right)}=0\;u_{tt}^{\left(2\right)}-c_{l}^{2}u_{aa}^{\left(2\right)}=\left(3c_{l}^{2}+C_{111}/\rho \right)u_{a}^{\left(1\right)}u_{aa}^{\left(1\right)}$$

Equations (4) and (5) are derived by using the perturbation method to obtain the solution of the first fundamental frequency and the second harmonic frequency, respectively. Here, $u^{\left(1\right)}$ represents the primary wave solution, and $u^{\left(2\right)}$ represents the second-order wave solution.

(4) $$u^{\left(1\right)}=A_{1}\exp j(k_{l}a-wt)$$

(5) $$u^{\left(2\right)}=A_{2}\exp j(2k_{l}a-2wt)$$

In the above equation, $A_{2}=\frac{\beta_{l}A_{1}^{2}k_{l}^{2}a}{{8C}_{l}^{2}}$, $\beta_{l}=\frac{A_{2}}{A_{1}^{2}}\frac{{8C}_{l}^{2}}{K_{l}^{2}x}$, where $\beta_{l}$ is the longitudinal second harmonic nonlinear parameter. $A_{1}$ and $A_{2}$ are the fundamental and second harmonic amplitudes, respectively. The nonlinear parameter is expressed as the ratio of the second harmonic amplitude divided by the square of the fundamental amplitude.

## 3. Experimental Setup

In order to investigate the nonlinearity of Lamb waves in response to bending stress, an experimental study was conducted on an aluminum plate (Al5052). The test specimens consisted of three specimens: one with no load applied to the same specimen, one with stresses in the elastic region below yield strength, and one with stresses in the plastic region above yield strength.

**Figure 1:**
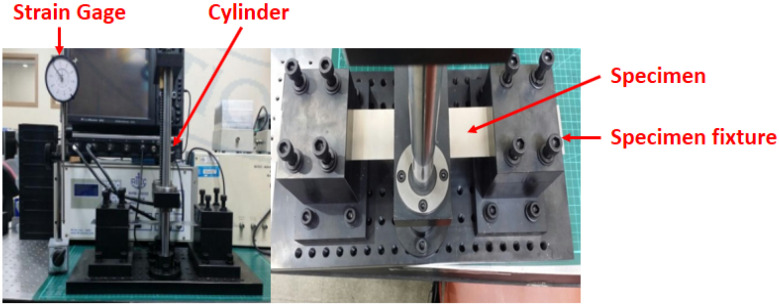
Bending device.

**Figure 2:**
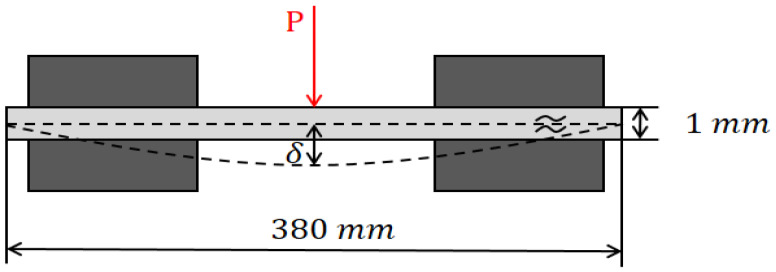
Application of load and corresponding deflected shape.

Figure 1 illustrates the fixation of the specimen to the equipment, and the relationship between maximum deflection and corresponding stress at the mid-span of the specimen was determined using a strain gauge. In order to fabricate the specimen, the aluminum material properties are referenced as shown in Table 1. The schematic representation of the application of load and corresponding deflected shape is depicted in Figure 2.

**Figure 3:**
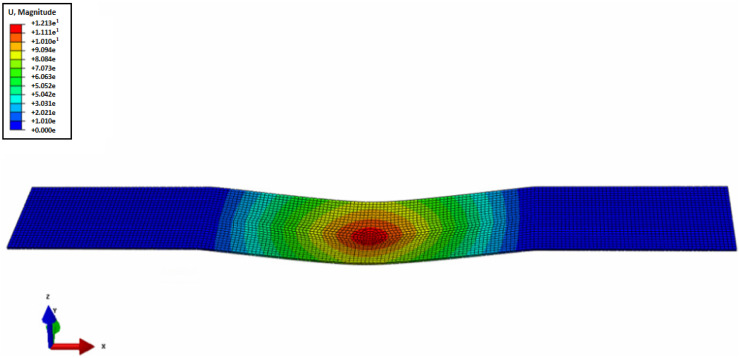
Displacement contour of the plate loaded beyond the elastic limit.

**Figure 4:**
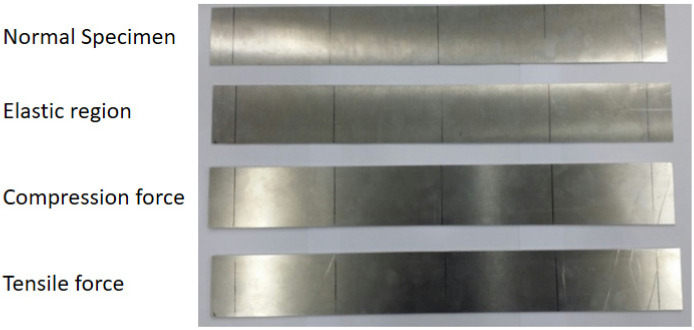
Aluminum specimens (Al5052).

Prior to conducting the experiment as shown in Figure 3, the deflection of the specimen was determined using the Structural Analysis Tool (ABAQUS 2021 ver., Dassault Systèmes, Vélizy-Villacoublay, France). This analysis aimed to ascertain the deflection value at the mid-span for both loading conditions: within the elastic limit and beyond the yield strength (plastic region). The structural analysis revealed that the plate remained in the elastic range until the maximum stress reached 40.5 MPa, with a corresponding maximum deflection of 9.45 mm. Based on these findings, experimental tests were conducted on the specimens. For the plate loaded within the elastic limit, a stress equal to half of the yield strength was applied. In contrast, for the plate loaded beyond the yield strength, a stress of 0.2% offset beyond the yield strength was applied. The stresses and their corresponding deflections in the elastic and plastic regions are presented in Table 2. Figure 4 visually represents the specimens before and after the bending test, capturing the bending damage incurred. Following the bending test, the specimens were utilized for nonlinearity measurements.

**Figure 5:**
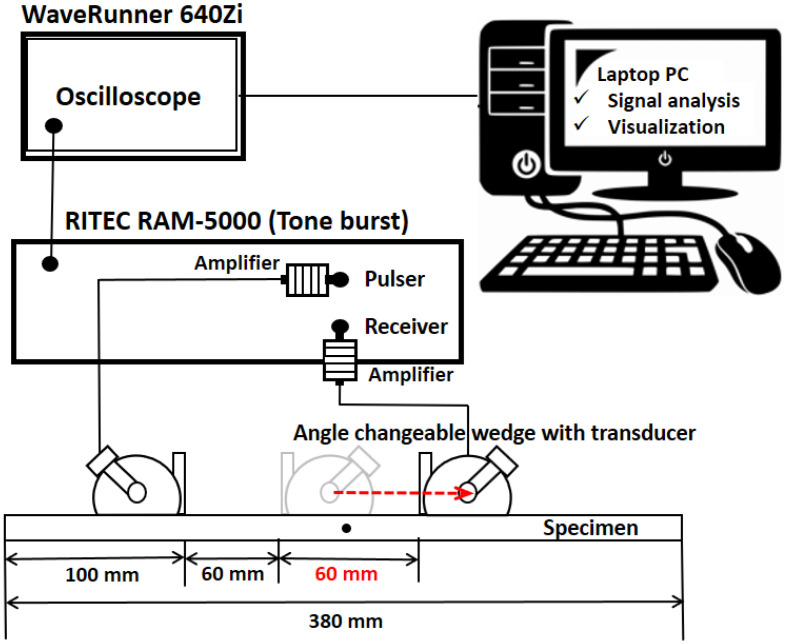
Schematic of experimental set up.

**Figure 6:**
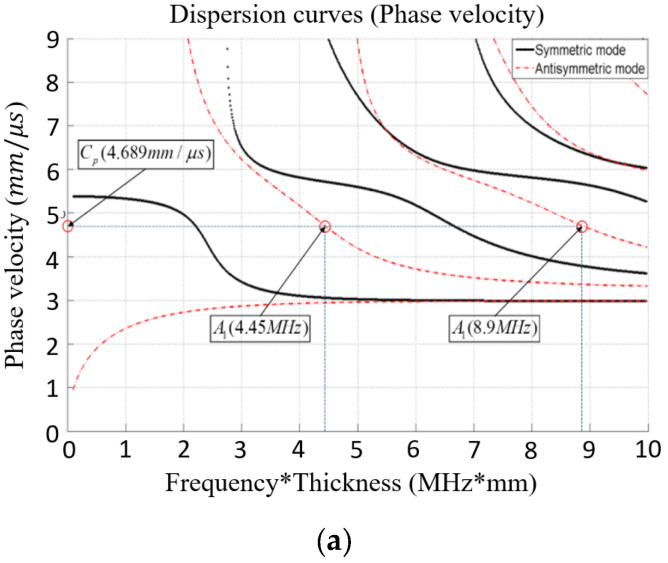

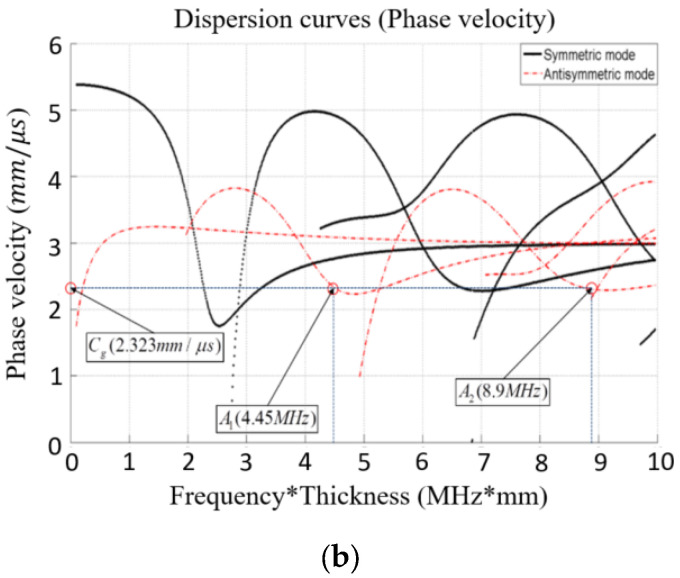
Mode selection: (**a**) Phase matching of antisymmetric mode ($A_{1}$); (**b**) Group velocity of antisymmetric mode ($A_{1}$).

A schematic representation of the nonlinear measurement system can be observed in Figure 5. The excitation signal is generated at a distance of 100 mm from the end of the specimen, and the signal was received at 10 mm intervals within a distance of 60 mm. The tone burst equipment (RAM-5000, RITEC Inc., Warwick, RI, USA) was employed for this purpose. The objective of this investigation was to explore the nonlinearity associated with bending stress. Therefore, particular attention was given to the region near the maximum deflection, where the signal reception was focused. Figure 6a,b show the phase matching of the antisymmetric mode, which was employed for frequency selection. Based on the velocity matching criteria of antisymmetric modes, the selected frequencies and their corresponding wave velocities are presented in Table 3 and Table 4.

**Figure 7:**
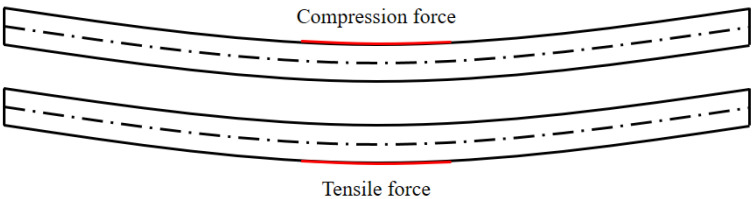
Compression and tensile force in specimen.

When stress is applied to the center of the plate, as illustrated in Figure 7, the upper surface of the plate experiences compressive forces while the lower surface undergoes tensile forces. It is well known that the tensile strength of materials is typically lower than their compressive strength. Consequently, it is expected that the lower surface, subjected to tensile forces, may exhibit a relatively higher occurrence of micro-defects or microstructure changes.

## 4. Experimental Result

In order to evaluate the presence of intrinsic material nonlinearity, initial nonlinear measurements were performed on an unstressed plate. To accomplish this, the nonlinearity of the specimen was measured in the antisymmetric mode, employing the same distance as described in the previous section (Figure 5).

**Figure 8:**
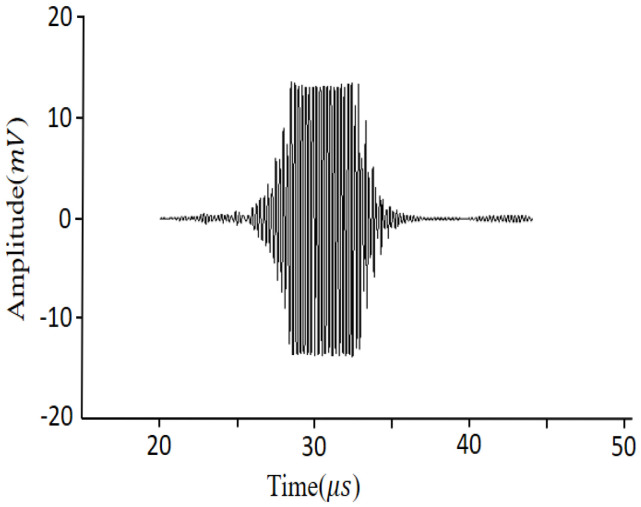
Source signal of $A_{1}$ mode (4.45 MHz) Lamb wave.

**Figure 9:**
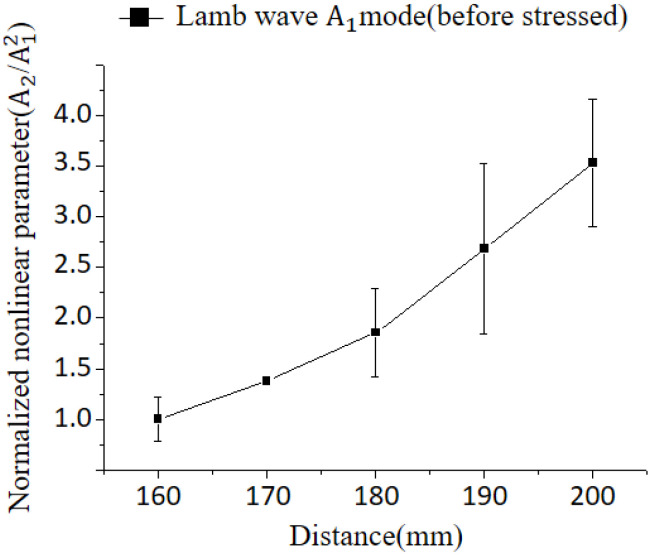
Nonlinear Lamb wave $A_{1}$ mode (4.45 MHz) applied to cumulative effect in Al5052 before stressed.

Figure 8 illustrates the received signal of the A1 mode Lamb wave, and the comparison of nonlinearity was conducted through the application of a fast Fourier transform to the signal. Figure 9 presents the variation of nonlinear parameters with respect to distance. The nonlinear parameter obtained for this stress-free state was set as the control, and the cumulative effect was also observed. Although considerable variations in the degree of nonlinearity were observed across different locations, these differences were not deemed statistically significant. Furthermore, it should be noted that the values depicted in the graph represent the relative size of the parameter. Thus, it is important to examine the trend within each interval rather than focusing solely on the absolute values.

**Figure 10:**
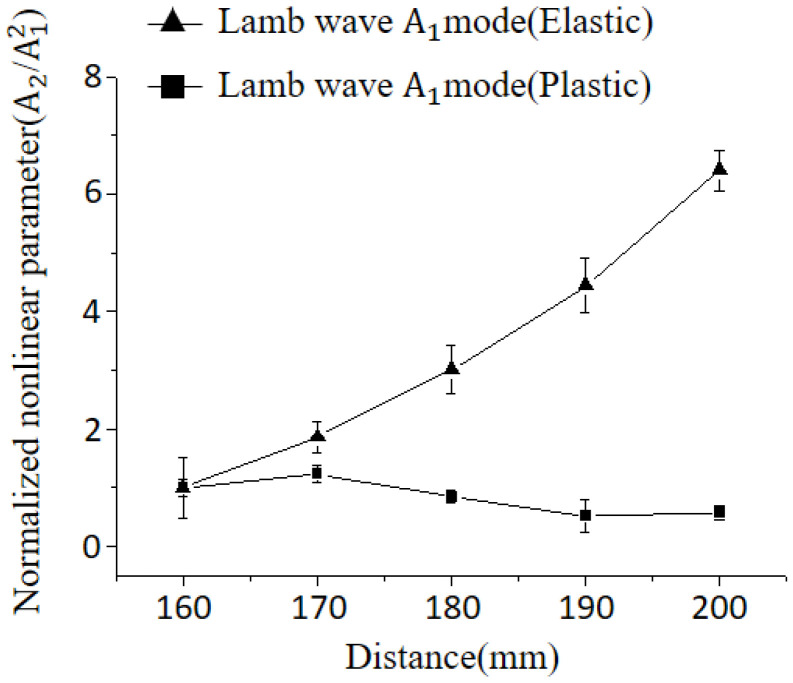
Comparison of nonlinear parameters for specimens after elastic recovery and plastic deformation (4.45 MHz).

Figure 10 presents the variation of the nonlinear parameter of the Lamb wave after elastic recovery, where a stress of 40.5 MPa was applied to the center of the specimen, as well as after plastic deformation at a stress of 89.6 MPa. The nonlinear characteristics exhibited distinct behaviors. Significant variations in the degree of nonlinearity were observed at different locations for the unstressed plate (Figure 9). In contrast, after elastic recovery, the variation in nonlinearity was comparatively reduced. However, nonlinear tendencies are dominantly influenced by the overlap effect, and it is difficult to find a tendency different from the previous one. For the specimen with plastic deformation, the nonlinear parameter exhibits a slight increase up to 70 mm, followed by a subsequent decrease. Beyond 90 mm, the parameter remains relatively constant.

**Figure 11:**
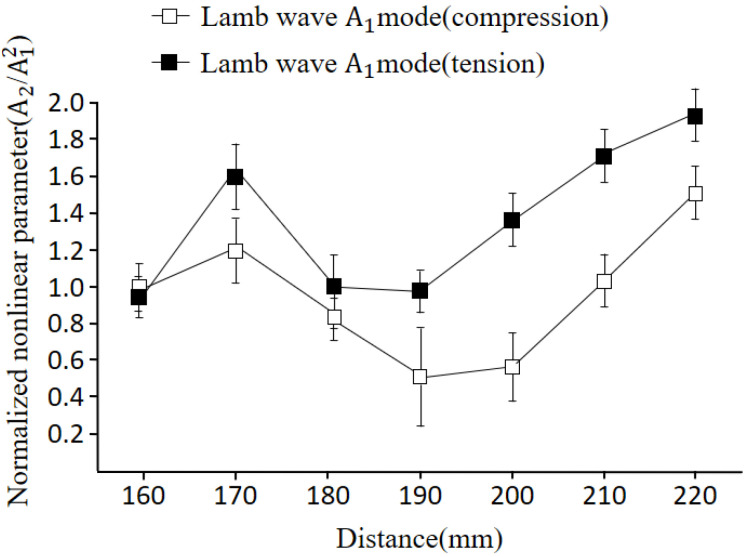
Comparison of nonlinear parameters due to compression and tensile stress (4.45 MHz).

The plate underwent bending, resulting in compressive stress on the upper surface and tensile stress on the bottom surface. To examine the influence of these stresses on nonlinearity, measurements were conducted at both the top and bottom surfaces. Figure 11 illustrates a graph comparing the nonlinear parameters attributed to compression and tensile stress.

Next, we investigated the dependences of nonlinearity for different Lamb wave modes on the specimen with plastic deformation. The upper surface (compression region) of the plate was scanned using the generated antisymmetric A1 mode and symmetric S1 mode.

**Figure 12:**
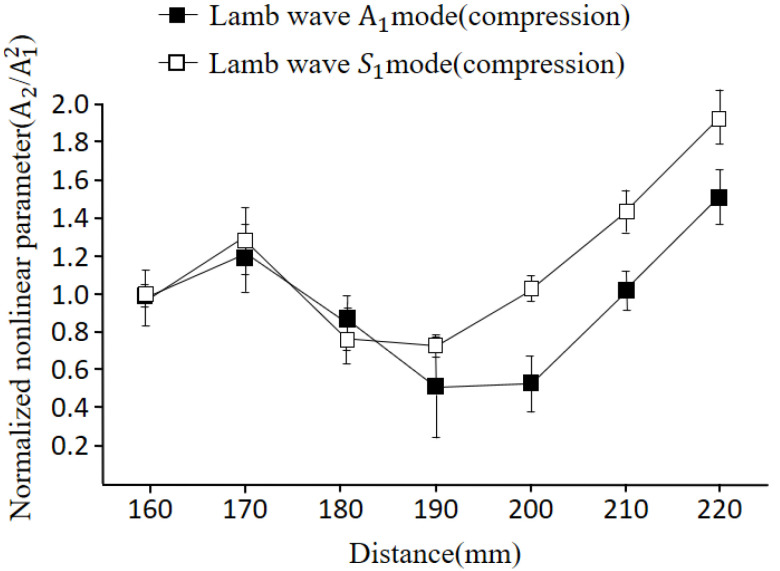
Comparison of nonlinearity between S1 and A1 modes for the upper surface of the plate (3.4 MHz).

**Figure 13:**
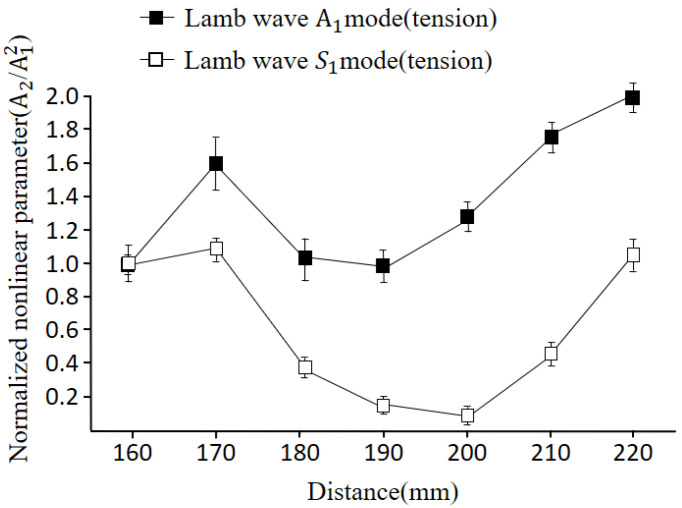
Comparison of nonlinearity between S1 and A1 modes for the bottom surface of the plate (3.4 MHz).

Figure 12 depicts a comparison between the antisymmetric mode and the symmetric mode. In both cases, the nonlinearities show an initial increase followed by a decrease beyond 70 mm, with a subsequent decrease up to 90 mm. The trend in the variation of the nonlinear parameter is similar for both modes, indicating no significant difference in in-plane or out-of-plane displacements. Figure 13 illustrates the comparison between the symmetric and antisymmetric modes for the bottom surface (tension region) of the plate. The trend in variation is similar to that shown in Figure 12. However, it can be observed that the nonlinear amplitude is relatively higher for the antisymmetric mode compared to the symmetric mode.

To precisely identify the location of plastic deformation, measurements were conducted at 2 mm intervals within the distance range of 70 mm to 80 mm. The measurements were performed using the antisymmetric Lamb wave mode. This approach aimed to capture the exact position of the plastic deformation, which exhibited a decreasing trend in nonlinearity between the 70 mm and 80 mm sections in the previous experiments.

**Figure 14:**
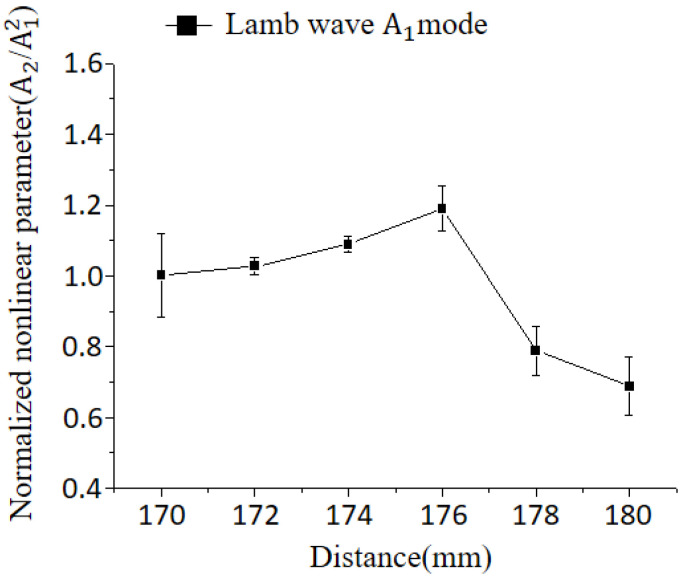
Variation of nonlinearity with distance, indicating maximum plastic deformation at 76 mm.

Figure 14 reveals that the nonlinearity exhibits an increasing trend up to 76 mm, followed by a subsequent decrease. This suggests that plastic deformation likely occurred in the 76 mm section. The decrease in nonlinearity can be attributed to signal scattering caused by the presence of plastic deformation.

## 5. Conclusions

In previous studies, nonlinear ultrasonic techniques have been employed to investigate the detection of micro-defects in materials and the correlation between nonlinearity and tensile/compressive loads. However, limited research has been conducted on the behavior of materials subjected to bending stress during the manufacturing stage, particularly for products with curvature created through bending rather than molds. This study aimed to examine the response of nonlinear Lamb waves to bending stress in aluminum plates.

Experimental investigations were conducted on specimens with both compressive and tensile stresses, representing the plastic deformation state. The nonlinear tendencies of Lamb waves were analyzed and compared, considering the dominant influence of the symmetric mode on in-plane displacement as a control group against the antisymmetric mode, which predominantly affects out-of-plane displacement. Through these experimental verifications, the following findings were obtained:

1. The nonlinearity of the specimens showed distinct behavior depending on the stress applied. Specimens subjected to stress within the elastic region exhibited increased nonlinearity, while specimens under stress within the plastic region displayed a decrease in nonlinearity. This indicates that the material’s response to bending stress varies depending on its elastic or plastic state;
2. When analyzing the specimens with plastic deformation, it was observed that the nonlinearity tended to decrease in sections experiencing both tensile and compressive forces. This suggests that the plastic deformation resulted in a reduction of the nonlinearity of the material, possibly due to signal scattering caused by the plastic deformation itself;
3. Both the symmetric and antisymmetric modes of Lamb waves exhibited similar nonlinear characteristics when subjected to compressive and tensile forces. This similarity suggests that the influence of stress on the nonlinearity of the material is consistent based on the chosen Lamb wave modes.

## 6. Discussion

Based on the aforementioned findings, the following deductions can be made. The nonlinearity of a material generally increases with the propagation distance of the ultrasonic waves. However, in the plastic deformation section, there is a decrease in nonlinearity followed by an increase in nonlinearity after the deformation. This decrease in nonlinearity can be attributed to amplitude attenuation or scattering caused by the deformation of the specimen’s shape. Additionally, microscopic changes in the structure of the specimen resulting from plastic deformation are likely to have an impact. The nonlinearity of specimens with plastic deformation, regardless of whether they experienced tensile or compressive stresses, showed a decrease. This consistent behavior across different modes indicates a high level of reliability in the test results.

Based on the aforementioned observations, it can be concluded that the utilization of the wave mixing technique, which offers superior sensitivity and scanning electron microscopy (SEM), can yield more accurate and meaningful results. Further experimental investigations are required to explore the changes in nonlinearity due to varying physical properties. This study provides valuable insights into the behavior of Lamb waves in response to bending stress.

## Figures and Tables

**Table 1:** Aluminum properties (Al5052).

$\mathrm{Yield}\;\mathrm{Stress}\;(\sigma_{Y})$	81.0 MPa (0.2% Offset)
Modulus of Elasticity (E)	70.3 GPa
Density (ρ)	2.68 g/cc
Poission’s ratio (ν)	0.33
Shear Modulus (G)	25.9 GPa

**Table 2:** Specimens information.

	Stress	Maximum Deflection	Thickness
Unstressed	-	-	1 mm
Elastic Region	40.5 MPa	9.45 mm	1 mm
Plastic region	89.6 MPa	12.13 mm	1 mm

**Table 3:** Frequency and velocity parameter in antisymmetric mode.

Mode	Antisymmetric
$A_{1}$ (Fundamental frequency)	4.45 MHz
$A_{2}$ (Second harmonic)	8.9 MHz
$C_{P}$ (Phase velocity)	4.689 mm/μs
$C_{S}$ (Group velocity)	2.323 mm/μs

**Table 4:** Frequency and velocity parameter in symmetric mode.

Mode	Antisymmetric
$S_{1}$ (Fundamental frequency)	4.45 MHz
$S_{1}$ (Second harmonic)	8.9 MHz
$C_{P}$ (Phase velocity)	6.035 mm/μs
$C_{S}$ (Group velocity)	4.510 mm/μs
